# Inhibition of miR-155 Promotes TGF-β Mediated Suppression of HIV Release in the Cervical Epithelial Cells

**DOI:** 10.3390/v13112266

**Published:** 2021-11-12

**Authors:** Jyotsna Gokavi, Sharwari Sadawarte, Anant Shelke, Urmila Kulkarni-Kale, Madhuri Thakar, Vandana Saxena

**Affiliations:** 1 Division of Immunology and Serology, Indian Council of Medical Research-National AIDS Research Institute, MIDC, Bhosari, Pune 411026, India; jyotsgokavi@gmail.com (J.G.); mthakar@nariindia.org (M.T.); 2 Bioinformatics Centre, Savitribai Phule Pune University, Pune 411007, India; sharwari.s.sadawarte@gmail.com (S.S.); anantshelkecell@gmail.com (A.S.); or urmila.kulkarni.kale@gmail.com (U.K.-K.)

**Keywords:** HIV, cervical, TGF-β, microRNA, host restriction factors, miR-155, target prediction

## Abstract

TGF-β has been shown to play a differential role in either restricting or aiding HIV infection in different cell types, however its role in the cervical cells is hitherto undefined. Among females, more than 80% of infections occur through heterosexual contact where cervicovaginal mucosa plays a critical role, however the early events during the establishment of infection at female genital mucosa are poorly understood. We earlier showed that increased TGF-β level has been associated with cervical viral shedding in the HIV infected women, however a causal relationship could not be examined. Therefore, here we first established an in vitro cell-associated model of HIV infection in the cervical epithelial cells (ME-180) and demonstrated that TGF-β plays an important role as a negative regulator of HIV release in the infected cervical epithelial cells. Inhibition of miR-155 upregulated TGF-β signaling and mRNA expression of host restriction factors such as *APOBEC-3G*, *IFI-16* and *IFITM-3*, while decreased the HIV release in ME-180 cells. To conclude, this is the first study to decipher the complex interplay between TGF-β, miR-155 and HIV release in the cervical epithelial cells. Collectively, our data suggest the plausible role of TGF-β in promoting HIV latency in cervical epithelial cells which needs further investigations.

## 1. Introduction

TGF-β is a multifaceted protein with myriad sets of functions, some of which include fibrosis, hematopoiesis and regulation of the immune response [1,2,3]. TGF-β, an immunosuppressive cytokine, is shown to be involved in HIV immunopathogenesis [4,5]. Different HIV proteins such as gp160, Tat and Vpu have been shown to induce TGF-β in NK cells, PBMCs and monocytic cells such as U937 [6,7,8]. Earlier studies have reported that TGF-β plays a dual role either by inhibiting HIV or by aiding to its infection depending on different cell types in a time-dependent and/or dose-dependent manner [9]. TGF-β has been shown to impede HIV infection either by downregulating HIV-LTR and upregulating host restriction factors or by suppressing HIV transcription in mammary cells, cells of macrophage lineage, primary monocyte derived macrophages (MDM), bronchial epithelial cells, etc. [1,9,10]. However, other studies have also reported that TGF-β enhanced the HIV infection in monocytes, primary mononuclear phagocytes in vitro [11] and in peripheral blood monocyte-derived macrophages by upregulation of HIV-1 LTR activity [9]. Additionally, TGF-β is also found to promote HIV latency by various pathways such as in bronchial epithelial cells, TGF-β suppressed the HIV release and promoted latency by upregulation of human host-restriction factor *BLIMP-1*. TGF-β mediated upregulation of *BLIMP-1* involved downregulation of miR-9-5p [10]. In another study, TGF-β-dependent T_EM_-T_CM_ differentiation has been shown to increase in latently infected T_CM_ cells after HIV-1 infection [12]. These data collectively suggest a differential and cell type specific role of TGF-β in HIV infection. However, its role in cervicovaginal mucosal cells during HIV infection is not well defined.

Among the females, more than 80% of HIV infections are attributed through heterosexual contact where cervicovaginal mucosal cells play critical role [13]. Factors that contribute to the extent of HIV acquisition and disease progression at the cervicovaginal mucosa (CVM) include structural integrity of female genital tract (FGT), host immune response as well as the stage of menstrual cycle [14,15,16]. Chronic inflammation in CVM has also been shown to promote HIV infection [17]. CVM is an immunologically distinct site as compared to peripheral blood and other mucosal surfaces such as gut mucosa and oral mucosa [6,18,19,20], hence it is important to understand the immune mechanisms involved at CVM during HIV infection. However, due to the lack of a suitable and feasible animal model and difficulty in obtaining sufficient number of cervical cells, the early events of HIV infection at the CVM are poorly understood. Most of the available data regarding the immune response at the CVM is gathered by cytokine profiling using cervicovaginal lavage (CVL) and/or immune profiling using the cytobrush samples of HIV infected women. Earlier we and others have shown the increased levels of various cytokines and chemokines such as CCL2, CXCL8, CXCL10, IL-1β, TNF-α, IL-8, IL-6, IL-10 and TGF-β in the CVL of HIV infected women [21,22,23,24,25]. We previously reported that among the elevated cytokines, TGF-β level was associated with increased cervical HIV shedding [24]. Taken together, these findings indicate the plausible role of TGF-β in viral shedding at the CVM, however the causal relationship is not yet established. Therefore, we investigated the role of TGF-β in cervical HIV release using an in vitro model. To further understand the molecular mechanism(s) involved, we examined the role of microRNA-155 in the regulation of TGF-β mediated response in cervical epithelial cells. MicroRNAs (miRs) are one of the key regulators of TGF-β responses. It has been found in SIV infection, miR-155 suppressed TGF-β mediated signaling in the oral mucosa which resulted in an increased immune activation [26]. Role of miR-155 in regulating TGF-β mediated responses has also been highlighted in other studies including chronic HCV infection [27,28,29,30,31].

In the present study, we demonstrated that TGF-β plays a potent negative regulator of HIV expression in the cervical epithelial cells. Further, neutralization of TGF-β promoted HIV release via upregulating miR-155. We also showed that inhibition of miR-155 in the cervical epithelial cells reduced HIV release by promoting TGF-β signaling and expression of host restriction factors such as *APOBEC-3G*, *IFI-16* and *IFITM-3*.

## 2. Materials and Methods

### 2.1. Cells and Virus Used for In Vitro Assays

Human cervical epithelial cell line, ME-180 (ATCC HTB-33, Manassas, VA, USA) was maintained in McCoy’s (modified) 5A medium (Cat # 16600082, Gibco, Thermo Fisher Scientific, Waltham, MA, USA) with 10% heat-inactivated fetal bovine serum (FBS) (Cat # 1614001, Gibco, Thermo Fisher Scientific, Waltham, MA, USA). Human T-lymphocyte Jurkat LTR-GFP CCR5+ (JLTRG-R5) (Cat # ARP-11586, NIH HIV Reagent Program, Manassas, VA, USA [32,33]) cells were grown in RPMI-1640 (Cat # 23400021, Gibco, Thermo Fisher Scientific, Waltham, MA, USA) supplemented with 10% FBS and were incubated at 37 °C with 5% CO_2_. Culture media for both the cell lines were supplemented with 100 U/mL penicillin and 100 µg/mL streptomycin. Human immunodeficiency virus−1 (HIV-1) infectious molecular clone pNL4.3 (Cat # ARP-114, NIH HIV Reagent Program, Manassas, VA, USA [34]) was transfected into HEK-293T (Cat # ARP-103, NIH HIV Reagent Program, Manassas, VA, USA) cells to prepare the NL4.3 HIV-1 virus stocks which was used in the assays. Briefly, the supernatant collected after 48 h was membrane filtered and titrated to determine TCID50.

### 2.2. Co-Culture of ME-180 with HIV-1 Infected T-Cells

JLTRG-R5 cells were infected with MOI 0.1 of HIV-1 virus for 4 h and washed 3 times to remove residual unbound virus particles, then incubated for 48 h. Infected cells were then co-cultured onto ME-180 cells. Prior to the co-culture, infected T cells were treated with 200 µg/mL Mitomycin-C (MMC) for 45 min. Briefly, 0.4 million ME-180 cells were seeded in 48-well plates in McCoy Media with 10% FBS and incubated overnight at 37 °C with 5% CO_2_ followed by addition of Mitomycin-C treated HIV-1 infected T cells at a ratio of 1:3. After 18 h of co-culture, ME-180 cells were washed, thoroughly, 3 times with serum-free media to remove T cells. At indicated time points, cells and supernatants were harvested for further assays. To confirm the absence of infected T-cells from the co-culture after Mitomycin-C treatment and washing, microscopic examination of the cells as well as staining of the cells both ME-180 and JLTRG-R5 (individually and in co-culture) with surface markers (CCR5 and CXCR4) using flow cytometry were performed. JLTRG-R5 cells express both the co-receptors CCR5 and CXCR4 unlike ME-180 cells.

### 2.3. TGF-β Neutralization in ME-180 Cells

Pan-specific TGF-β neutralization antibody (NAb) (Cat # AB-100-NA, R&D Systems, Minneapolis, MN, USA) was added to ME-180 cultures (15 µg/mL) and kept for 18 h. Post 18 h, HIV-1 infected JLTRG-R5 cells were overlaid onto pre-treated ME-180 culture and infection was carried out as mentioned above. Cells were further supplemented with 10 µg/mL TGF-β NAb.

### 2.4. Transfection of miR-155 Inhibitor in ME-180 Cells

ME-180 cells were transfected with 100 nM of miR-155 inhibitor (Cat # IH-300647-06-0020, Dharmacon, Lafayette, CO, USA) or scrambled miR inhibitor control (Cat # IN-001005-01-20, Dharmacon, Lafayette, CO, USA) with rapid transfection protocol by Qiagen HiPerfect kit (Cat # 301704, Hilden, Germany) as per manufacturer’s instructions. After 18 h, cells were replenished with fresh media and treated with TGF-β NAb for an additional 18 h, and then co-cultured with HIV-1 infected JLTRG-R5. The cells were harvested for total RNA isolation and subsequently used for real-time PCR assay while supernatant was used to quantitate HIV-1p24 and TGF-β protein by ELISA.

### 2.5. Total RNA Isolation, cDNA Synthesis and Quantitative Real-Time PCR (q-PCR)

Total RNA was extracted from the harvested cells using TRI Reagent solution (Cat # AM9738, Invitrogen, Thermo Fisher Scientific, Waltham, MA, USA) and stored at −70 °C until further use. Complementary DNA (cDNA) synthesis for mRNA expression analysis was performed using manufacturer’s instruction provided with PrimeScript™ RT Reagent Kit (Perfect Real Time) (Cat # RR037B, Takara Bio Inc., Kusatsu, Shiga, Japan). Expression of mRNA was determined by performing real-time PCR using PowerUp™ SYBR™ Green Master Mix (Cat # A25742, Applied Biosystems™, Thermo Fisher Scientific, Waltham, MA, USA) on the 7500 Fast Real-Time PCR System (Applied Biosystems™, Thermo Fisher Scientific, Waltham, MA, USA) using primers (Integrated DNA Technologies, Coralville, IA, USA). The genes accessed were, *β-actin* (Fq-5′-TCGTCCACCGCAAATGCTTCTAG-3′, Rq-5′-ACTGCTGTCACCTTCACCGTTCC-3′), *TGF-β* (Fq-5′-CCCAGCATCTGCAAAGCTC-3′, Rq-5′-GTCAATGTACAGCTGCCGCA-3′), *GAG* (Fq-5′-ACCCATGTTTACAGCATTATCAGA-3′, Rq-5′-(GCTTGATGTCCGCCTACTGTATTT-3′), *SMAD-2* (Fq-5′-ACCGAAATGCCACGGTAGAA-3′,Rq-5′-TGGGGCTCTGCACAAAGAT-3′), *SMAD-3 (*Fq-5′-TGGGGCTCTGCACAAAGAT-3′, Rq-5′-TTGCCCTCATGTGTGCTCTT-3′), SMAD-4 (Fq-5′-CATCCTGCTCCTGAGTATTGG-3′, Rq-5′-GGGTCCACGTATCCATCAAC-3′), *SMAD-5* (Fq-5′-CTGGGATTACAGGACTTGACC-3′, Rq-5′-AAGTTCCAATTAAAAAGGGAGGA-3′), *APOBEC-3G* (Fq-5′-CGCAGCCTGTGTCAGAAAAG-3′, Rq-5′-CCAACAGTGCTGAAATTCGTCATA-3′), *IFI-16* (Fq-5′-CCCAAAGAAGATCATTGCCATAG-3′, Rq-5′-GTTTCGGTCAGCATTCACATC-3′), *IFITM-1* (Fq-5′-AGCACCATCCTTCCAAGGTCC-3′, Rq-5′- TAACAGGATGAATCCAATGGTC-3′), *IFITM-3* (Fq-5′-ATGAATCACACTGTCCAAACCTTCT-3′, Rq-5′- CTATCCATAGGCCTGGAAGATCAG-3′). Gene expressions were normalized to the β-actin mRNA. For determining microRNA (miR) expression, cDNA from total RNA was synthesized by TaqMan™ MicroRNA Reverse Transcription Kit (Cat # 4366596, Applied Biosystems™, Thermo Fisher Scientific, Waltham, MA, USA) and TaqMan™ MicroRNA assays for miR-155 and RNU-44. Real-time PCR was performed by TaqMan™ Universal Master Mix II, no UNG (Cat # 4,440,040 Applied Biosystems™, Waltham, MA, USA) using pre-validated TaqMan™ primer-probes for mir-155 and RNU-44 (Assay ID: 002,623 and 001,094 respectively, Applied Biosystems™, Waltham, MA, USA) according to the manufacturer’s protocol. The level of expression of the miR-155 gene was normalized to the RNU-44 gene.

### 2.6. ELISA

TGF-β and HIVp24 proteins were quantitated by using ELISA. TGF-β levels and HIVp24 levels from harvested supernatants were determined using Human TGF-β 1 DuoSet ELISA (Cat # DY240, R&D Systems, Minneapolis, MN, USA) and HIV-1 p24 Antigen Capture Assay (Cat # 5447, ABL. Inc., Rockville, MD, USA), respectively, according to the manufacturer’s instructions.

### 2.7. miRNA-mRNA Target Prediction

miRNA-mRNA target prediction was carried out using bioinformatics tools. The 3′ UTR sequences of the genes *APOBEC-3G*, *IFI-16* and *IFITM-3* were extracted from UCSC browser genome build GRCh38 (https://genome.ucsc.edu/; accessed on 8 November 2021) [35]. The miRNA sequences of miR-155 were extracted from miRBase (https://www.mirbase.org/; accessed on on 8 November 2021) [36]. RNA hybrid server (https://bibiserv.cebitec.uni-bielefeld.de/rnahybrid; accessed on on 8 November 2021) was used to predict miRNA-mRNA interactions with seed match of 2–8 bp [37,38].

### 2.8. Statistics

Experiments were repeated 2–3 times in triplicates. GraphPad Prism software version 5.0 was used for plotting graphs and performing statistical analysis. Unpaired Student’s *t*-test was used to determine the level of significance between different conditions, unless otherwise mentioned. A *p* value of ≤ 0.05 was considered significant.

## 3. Results

### 3.1. Neutralization of TGF-β in the Cervical Cells Increases HIV Release

**Figure 1:**
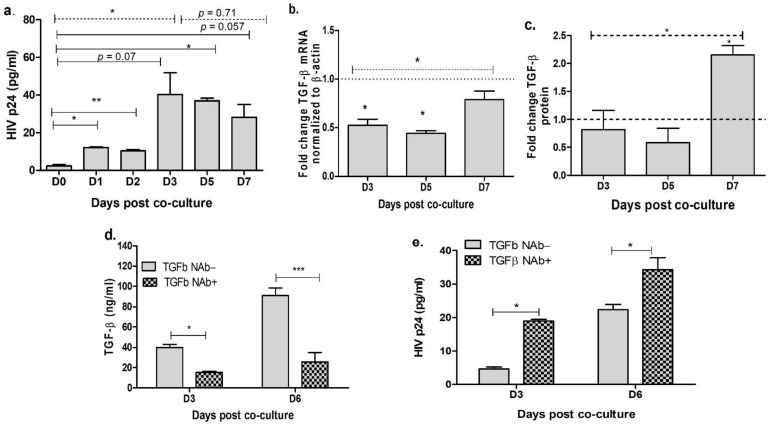
Neutralization of TGF-β in the cervical cells increases HIV release. JLTRG-R5 cells were infected with HIV NL4.3 (MOI = 0.1) for 48 h and co-cultured with ME-180 cells after Mitomycin-C treatment. At indicated time intervals, cells and supernatant were harvested. Supernatant was used to quantitate HIVp24 levels by ELISA (**a**). From cells RNA was extracted to quantitate TGF-β mRNA using real-time PCR. Fold change expression was calculated over the uninfected cells (baseline) which is shown as dotted line (**b**). TGF-β protein levels were determined in the supernatant using ELISA and fold change of infected cells was calculated over uninfected cells (**c**). ME-180 cells were treated with TGF-β neutralizing antibodies (NAb) for 18 h followed by co-culture with HIV infected JLTRG-R5 cells. TGF-β levels (**d**) and HIV release (**e**) was determined using ELISA at day 3 and day 6. Dotted line indicates one-way Anova analysis; solid lines indicate statistical analysis performed by unpaired Student’s *t*-test. Significance was determined by *p* value < 0.05.* indicates *p* value < 0.05, ** indicates *p* value < 0.01 and *** indicates *p* value < 0.001.

Cervical epithelial cells do not possess the receptors such as CD4+ and CXCR4/CCR5 for HIV-1 entry, and hence these cells are not susceptible for direct infection. However, these cells are shown to get infected via cell–cell associated route. To establish in vitro model for HIV infection in the cervical epithelial cells, we co-cultured NL4.3 infected JLTRG-R5 T-cells with ME-180 cell using the modified protocol as described earlier by Phillips et al. [39]. Absence of T cells after mitomycin treatment and washing was confirmed using microscopic examination (Supplementary Figure S1a) and absence of CCR5 and CXCR4 in the ME-180 cultures post washing (Supplementary Figure S1b) indicates the absence of T cells. Culture supernatants were collected at different time intervals and HIV-1 p24 proteins were quantitated by ELISA to examine HIV release. We observed that HIVp24 levels were significantly increased at 1, 3 and 5 days post-co-culture (*p* < 0.05) when compared with day 0. HIVp24 levels remained increased till day 6 (*p* = 0.057; Figure 1a). Among the infected cells, using one-way Anova analysis our results showed a significant change in HIVp24 expression till day 3 (*p* = 0.04), which remained unchanged at later time points (*p* = 0.71) (Figure 1a). As we have earlier shown that TGF-β level was associated with cervical viral load in HIV infected women [24], we next determined the expression of TGF-β mRNA and protein in ME-180 cells post co-culture with infected T cells. Interestingly we found a moderate but significant decrease in the expression of TGF-β mRNA at days 3 and 5 (Figure 1b) in the infected cells than the uninfected cells (*p* = 0.02 and 0.03, respectively) which increased at day 7 (*p* = 0.02). In contrast to mRNA expression, no significant difference was found in TGF-β protein at days 3 and 5 while a significant increase in TGF-β (*p* = 0.02) was noted at day 7 post co-culture with infected cells (Figure 1c). One-way Anova analysis showed an overall increase in both TGF-β mRNA expression and protein from day 3 to day 7 in HIV infected cells (Figure 1b,c). Since an overall increase in TGF-β expression was noted in HIV infected co-culture, we next sought to determine the effect of TGF-β neutralization on HIV release. ME-180 cells were treated with 15 µg/mL of TGF-β NAb for 18 h prior to co-culture with infected T cells. We confirmed the effect of TGF-β neutralization using ELISA and found that TGF-β levels remain suppressed throughout the assay in the ME-180 cells treated with TGF-β NAb than the untreated cells (Figure 1d). Our results showed that TGF-β neutralization significantly increased the expression of HIV p24 at both 3 days (*p* = 0.05) and 6 days (*p* = 0.03) post co-culture with the infected T cells as compared to the non-neutralized cells (Figure 1e). These results indicate that depletion of TGF-β enhances the HIV release in the cervical epithelial cells. However, it is unclear how depletion of TGF-β enhances the expression and release of HIV protein in the cervical epithelial cells.

### 3.2. Inhibition of miR-155 Increases TGF-β Signaling in the Cervical Epithelial Cells

**Figure 2:**
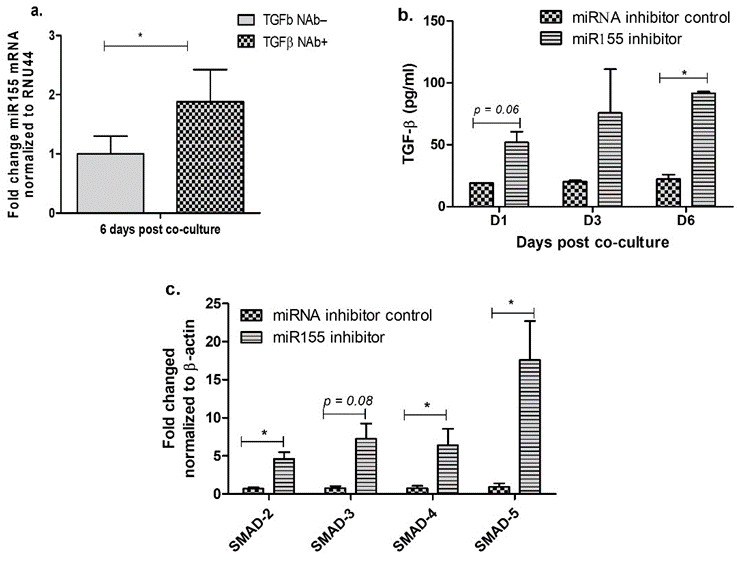
Inhibition of miR-155 increases TGF-β signaling in the cervical epithelial cells. Assessing miR-155 gene expression in the HIV infected ME-180 cells in the presence and absence of TGF-β NAb (**a**). ME-180 cells were transfected with miR-155 inhibitor or scrambled miR inhibitor (mock) 24 h prior to co-culture with the infected T cells and at indicated time intervals, cells and supernatant were collected. Supernatant was used for the quantitation of TGF-β protein by ELISA (**b**); and cells were used to determine the gene expression of SMAD-2, SMAD-3, SMAD-4, and SMAD-5 using real-time PCR. Fold change mRNA expression was calculated over mock transfected cells (**c**). Statistical analysis was performed by unpaired Student’s *t*-test to determine the level of significance. * indicates *p* value < 0.05.

In order to find out the mechanism on how depletion of TGF-β enhances the expression and release of HIV protein in the cervical epithelial cells, we investigated the role of miR-155 in TGF-β mediated HIV release. miRs are one of the key regulators of TGF-β signaling [30], and earlier studies have shown that miR-155 has specific targets in TGF-β signaling pathway [28,30]. To determine if miR-155 levels are altered during TGF-β neutralization, we quantitated the expression of miR-155 in TGF-β neutralized cells. Interestingly, we found a significant increase in miR-155 expression in TGF-β neutralized ME-180 cells than the non-neutralized cells (Figure 2a). To further understand the relationship between TGF-β and miR-155, ME-180 cells were transfected with miR-155 inhibitor or scrambled inhibitor (mock) prior to treatment with TGF-β neutralizing antibodies. We validated the depletion of miR-155 upon treatment with miR-155 inhibitor (Supplementary Figure S2). We found that compared with cells treated with scrambled inhibitor (mock), cells treated with miR-155 inhibitor showed an increased release of TGF-β protein at day 1 (*p* = 0.06) and day 6 (*p* = 0.044) (Figure 2b). Since TGF-β signaling is mediated through *SMADs* [40,41,42], and it is shown that increased miR-155 levels suppress *SMAD-5* in the oral mucosa [26], we next examined the expression of *SMAD* genes in the cells transfected with miR-155 inhibitor. Increased expression of *SMAD-2* (*p* = 0.01), *SMAD-4* (*p* = 0.04). *SMAD-5* (*p* = 0.03) and *SMAD-3* (*p* = 0.08) mRNA was observed in ME-180 cell transfected with miR-155 inhibitor when compared with the cells treated with scrambled (mock) inhibitor (Figure 2c). These results showed that inhibition of the miR-155 in the cervical epithelial cells increases the TGF-β mediated signaling during HIV infection.

### 3.3. Inhibition of miR-155 Increases the Expression of Host Restriction Factors and Suppresses HIV Release in the Cervical Epithelial Cells

**Figure 3:**
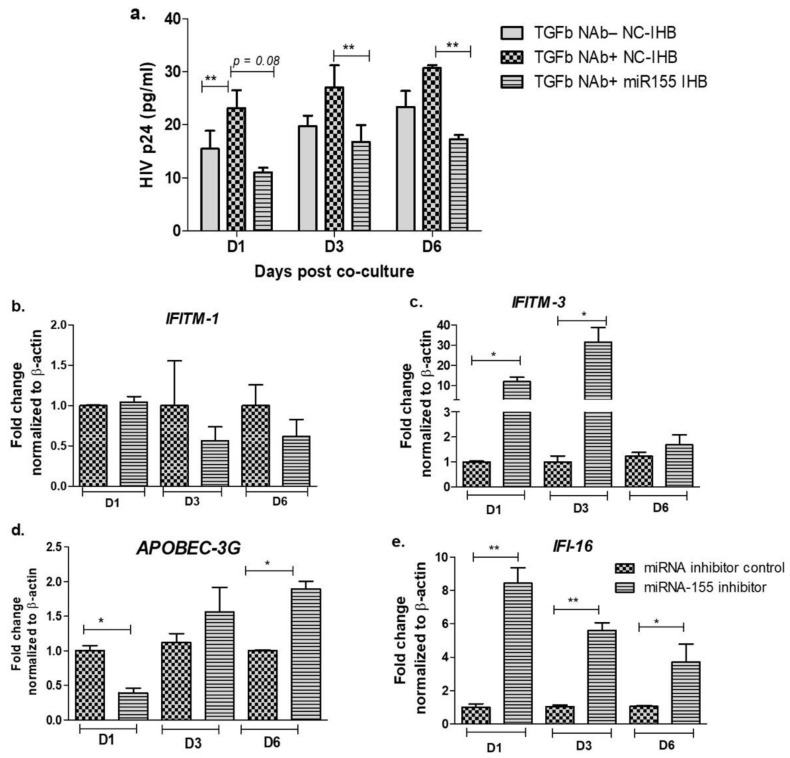
Inhibition of miR-155 increases the expression of host restriction factors and suppresses HIV release in the cervical epithelial cells. ME-180 cells were transfected with miR-155 inhibitor or scrambled miR inhibitor (mock) for 24 h followed by neutralization of TGF-β with TGF-β NAb and then co-cultured with HIV infected JLTRG-R5 cells. At the indicated time points, supernatants were harvested and quantitated for HIVp24 using ELISA (**a**). Cells treated with mentioned conditions were used to examine the mRNA expression of host restriction factors such as *IFITM-1* (**b**)*, IFITM-3* (**c**)*, APOBEC-3G* (**d**) and *IFI-16* (**e**) using real-time PCR. Fold change mRNA expression normalized to β-actin was calculated over mock. Statistical analysis was performed by unpaired Student’s *t*-test. * indicates *p* value < 0.05 and ** indicates *p* value < 0.001.

Our data indicate that reduced level of miR-155 increases the expression of TGF-β and its downstream signaling molecules such as SMADs (Figure 2) and neutralization of TGF-β increases the HIV release (Figure 1). We next wanted to investigate whether miR-155 is involved in HIV release in the cervical epithelial cells. We found that although TGF-β neutralization increased virus release in the cell treated with scrambled miR inhibitor (Figure 3a), prior blocking of miR-155 expression significantly decreased the HIVp24 expression at days 3 (*p* = 0.009) and 6 (*p* = 0.006) (Figure 3a). Although a reduction of HIVp24 was observed at day 1, it did not reach to the level of significance (*p* = 0.08). To elucidate whether treatment with TGF-β neutralizing antibodies and miR-155 inhibitor influences viral gene expressions or specifically targets the viral release, we examined the HIV gag mRNA expression. We found a significant reduced expression of HIV Gag mRNA in the treated cells compared to the mock treated cells at days 3 and 6 (*p* = 0.05 and *p* = 0.006, respectively). At day 1 however, no significant difference could be noted but there is reduced trend of Gag mRNA expression in the cells treated with miR-155 inhibitor and TGF-β neutralizing antibodies (Supplementary Figure S3). These results indicate that miR-155 interferes with the TGF-β mediated suppression of HIV gene expression and release. We also examined the effect of miR-155 treatment alone on HIV release. Interestingly it was observed that at days 1 and 3, there is a significant reduction in HIV-1 release in the miR-155 inhibited cells than the mock controls (scrambled inhibitor), but not at day 6 post infection. Although a reduced trend of HIV release was noted at day 6 in the miR-155 inhibitor treated cells (Supplementary Figure S4). It may be likely due to modest restrictive effect of miR-155 inhibitor on the viral release, unlike nearly 4-fold increase in TGF-β level at this time point i.e., day 6 (Figure 2b).

Since, host restriction factors hamper the HIV replication and its release, we further examined whether miR-155 inhibition alters the gene expression of the HIV-1 viral protein synthesis (*IFITM-1* and *IFITM-3*) [43], reverse transcription (*APOBEC-3G*) [44,45,46] and HIV gene expression (*IFI-16*) [47,48]. While there was no significant change in the expression of *IFITM-1* mRNA (Figure 3b) as a result of miR-155 inhibition, we found a significant increase in the expression of *IFITM-3* gene at days 1 (11-fold; *p* = 0.03) and 3 (31-fold; *p* = 0.01) post infection in the ME-180 cells transfected with miR-155 inhibitor as compared to the mock cells (Figure 3c). While at day 1 post infection, the *APOBEC-3G* gene was significantly reduced in the miR-155 knocked down cells, there was a significant increase in *APOBEC-3G* mRNA in the ME-180 cells transfected with miR-155 inhibitor at 6 days post infection (2-fold; *p* = 0.01) than the mock transfected cells (Figure 3c). Further cells treated with miR-155 inhibitor showed a significant increase in the expression of *IFI-16* gene (*p* < 0.05) than the cells transfected with miR-inhibitor control/mock (Figure 3e). Since we found that inhibiting miR-155 in cervical epithelial cells increased the gene expression of *APOBEC-3G, IFI-16* and *IFITM-3*, we next sought to determine whether miR-155 can directly target the mRNA of these restriction factors by using RNA hybrid prediction algorithm. Multiple miRNA-mRNA hybridization sites were detected for all the genes of interest i.e., *APOBEC-3G, IFI-16 and IFITM-3*. The most optimum target site for every gene was selected on the basis of minimum free energy (mfe). The top-ranking binding sites for the genes of interest are listed in Table 1. The observed binding targets of miR-155 for the genes of interest viz., *APOBEC-3G, IFI-16* and *IFITM3* thus indicate the potential role of these miR-155 in the regulation of expression of the target genes. Collectively, these findings indicate that inhibition of miR-155 increases the expression of host restriction factors such as *APOBEC-3G, IFI-16* and *IFITM-3* in the ME-180 cells, which could result in suppression of the HIV shedding.

## 4. Discussion

Among the females, cervicovaginal mucosa plays critical role in acquiring HIV infection during heterosexual contact. However, unlike the peripheral system and other mucosal surfaces, the immune mechanisms involved in the cervical mucosa are poorly understood. Since genital mucosa are immunologically distinct sites [15,49], it becomes imperative to understand the host immune factors at these local sites. Limited studies are available due to unavailability of suitable small animal models, challenges not only in collecting the clinical samples but also limited cervical cells obtained to examine kinetic studies, etc. Hence, we first attempted to establish in vitro model of HIV infection in cervical epithelial cells as reported previously by Phillips et al. with minor modifications [39]. Absence of HIV entry receptors on the cervical cells makes them unsusceptible cells for direct HIV infection [50], however cell to cell associated transmission such as transcytosis has been shown to establish HIV infection in the cervical epithelial cells in vitro [39,51,52,53,54]. We demonstrated the infection of ME-180 cells by co-culturing the HIV infected T cells (JLTRG-R5) and quantitating the release of HIVp24, validating the usefulness of this in vitro model to study HIV infection in the cervical cells. We observed a significant increase in HIV release in the ME-180 cells till day 3 post co-culture with infected T cells which remained unchanged afterwards, suggesting that HIV release by the cervical epithelial cells hampers at later time post co-culture with HIV infected T cells. Given that, the levels of TGF-β protein are increased in the CVL of HIV infected women [23,24], and its correlation with higher cervical viral load [24], we next examined the role of TGF-β in HIV release by the cervical cells. We found decreased TGF-β mRNA expression at days 3 and 5 in ME-180 cells post co-culture with infected T cells compared to the uninfected co-cultures, while no significant difference in TGF-β protein level was observed. This apparent anomaly could be related to the transcriptional and translational difference of TGF-β, however the TGF-β expression was increased at day 7. Our results suggest that increased TGF-β might play a role in abrogating HIV infection and its release in the cervical epithelial cells. This observation contradicts the previous findings where increased TGF-β has been shown to be associated with cervical virus shedding [22,24]. In one of these reports, it was suggested by Gumbi et al. that TGF-β had a negative impact on the local immune responses and helped in maintaining pro-HIV replicative environment at the cervical mucosal level in HIV-infected women [22]. The likely reason for these contradictory findings in the present and previous studies could be the different samples that were tested for estimation of TGF-β and HIV release. Infected cervical epithelial cells under in vitro condition were used in the present study while other studies have estimated the TGF-β levels in the cervicovaginal lavage from HIV infected women, where the source of TGF-β as well as HIV could be attributed through other immune cells such as regulatory T cells and other T cell subsets as well.

TGF-β has been shown to negatively regulate HIV expression [1,10], hence we examined to decipher the role of TGF-β in HIV release by the cervical epithelial cells using TGF-β neutralizing antibodies. TGF-β neutralization in cervical epithelial cells resulted in an increased expression of HIVp24 protein in comparison with the non-neutralized cells, suggesting that TGF-β might be involved in restricting HIV release in the cervical epithelial cells. Our observations are in corroboration with the previous findings where increased TGF-β has been shown in restricting HIV release and promoting latency in bronchial epithelial cells [10], in cells of monocytic/macrophage lineage [1] and also in mammary epithelial cells [9]. Hence, collectively our findings and others suggest that TGF-β is involved in restricting HIV release and thus might play a potent role in promoting HIV latency in various cell types. During HIV latency, the expression and release of HIV-1 proteins are largely hampered, and different latency reversal agents have been shown to reactivate latently infected cells by quantitating the levels of HIV-1p24 protein [55,56,57]. Since cervical tissues have also been shown to harbor latent infection [49,58,59,60], our findings indicate a plausible role of TGF-β in maintaining latency and open avenues for more investigations. It would be of interest to examine the potential of TGF-β overexpression on HIV release and TGF-β blockers to reactivate the latently infected cells. One of the limitations of our study is that we have used TGF-β neutralizing antibodies, however additional targeting of TGF-β by multiple siRNAs/shRNAs and examining its effect on virus release in the TGF-β depleted cells could be an interesting question for future studies.

TGF-β being a pleiotropic cytokine exerts multiple effects on different cell types through various signaling pathways. MicroRNAs (miRs) are one of the key regulators of TGF-β signaling [30] and various microRNAs have been shown to target different components in the TGF-β signaling. Among others, miR-155 is well reported to regulate and alter TGF-β signaling in viral diseases, immune function as well as in various cancers [28,61,62,63,64]. Further it has been described that T cells infected with HIV disseminate miR-155 to the cervical cells via cell-derived exosomes [29]. Hence, we ought to determine the relationship between TGF-β and miR-155 in cervical epithelial cells during HIV infection. TGF-β neutralization resulted in a two-fold increase in miR-155 gene expression when compared with the non-neutralized cells. To further understand the relationship between miR-155 and TGF-β, cells were treated with miR155 inhibitor which showed a significant increase in TGF-β. Given that, binding of mature TGF-β to TGF-β1 receptor activates TGF-β signaling resulting in activation of downstream molecules, including SMAD family transcription factor complexes [65] and the role of miR-155 in targeting TGF-β RII, SMAD-2 and SMAD-5 [30,31], we next found that inhibition of miR-155 in cervical cells resulted in a significant increase in gene expression of SMADs- 2, 3, 4 and 5 than those treated with scrambled miR inhibitor. These results confirm that TGF-β mediated signaling is altered by miR-155 in the cervical epithelial cells during HIV infection. Similar to our observation, previously a higher level of miR-155 has also been shown to dysregulate TGF-β signaling in the oral mucosa during chronic SIV infection [26].

We next analyzed the probable role of miR-155 in HIV release by the cervical epithelial cells and found that while TGF-β neutralization increased the HIV shedding, inhibition of miR-155 prior to TGF-β neutralization restricted the virus release as well as viral gene expression by the cervical epithelial cells. Further, since host restriction factors interfere with the HIV replication and its release, we examined whether inhibition of miR-155 alters the gene expression of the cellular restriction factors affecting, HIV-1 viral protein synthesis (*IFITM-1* and *IFITM-3*) [43], reverse transcription (*APOBEC-3G)* [44,45,46] and HIV gene expression (*IFI-16*) [47,48]. We found that except *IFITM-1*, the expression of *IFITM-3*, *APOBEC-3G* and *IFI-16* genes were increased in the presence of miR-155 inhibitor. In miRNA-mRNA target prediction analysis, we found the observed binding targets of miR-155 for *APOBEC-3G, IFI-16* and *IFITM-3* which further indicates the potential role of miR-155 in the regulation of these host restriction factor genes during HIV infection. APOBEC family of proteins are known to provide intrinsic innate immunity against retroviruses like HIV in the host cells, wherein they affect the HIV replication [45]. APOBEC-3A has been shown to suppress HIV-1 proviral transcription by binding to the LTR region and HIV-1 p24 release by the latently infected cells subsequently promote HIV latency [66]. Interferon induced transmembrane proteins (IFITMs) 1, 2 and 3 are anti-viral factors which inhibit viral protein synthesis into the cells by targeting the viral mRNA and halting HIV-1 translation [43]. Different IFITM proteins have been shown to vary in their inhibitory effect depending on HIV-1 co-receptor usage and cell type. Lee et al. have shown HIV entry independent effect, where IFITM-1 and IFITM-2 were found more restrictive than IFITM-3 in HEK293T cells while inducible expression of IFITM-3 showed an equivalent antiviral effect to IFITM-1 and IFITM-2 in SupT1 cells [43]. Other studies have shown that while IFITM-1 inhibits the entry of CCR5-tropic viruses, IFITM-2/3 predominantly inhibits CXCR4-tropic viruses [43,67,68]. Our data is in consistency with the latter reports and suggest that IFITM-3 and not IFITM-1 might be important in restricting HIV release after miR-155 inhibition in the ME-180 cells. It could be largely due to the fact that we have used HIV-1 NL4.3 virus strain which is a CXCR4 tropic strain. Interferon-γ-inducible protein 16 (IFI-16), a member of the pyrin and HIN domain (PYHIN) containing protein family acts as an intracellular immune sensor of the dsDNA in the cytosol of the infected cells resulting to launch either a IFN or inflammasome mediated immune response to HIV, HCMV and HSV-1 [47]. Thus, all these restriction factors have been shown to constrain HIV replication which ultimately results in reduced HIV release. Previously microRNAs such as let-7c, miR-34a or miR-124a have been shown to increase HIV release by downregulating the cellular restriction factors—p21 and TASK1 [69]. In the present study, our findings indicate that inhibition of miR-155 in the ME-180 cells resulted in increased expression of the host restriction factors *APOBEC-3G, IFI-16* and *IFITM-3* which may have restricted the viral replication and hampered the HIV release by the cervical epithelial cells. However, the direct role of these restriction factors needs further investigations.

**Figure 4:**
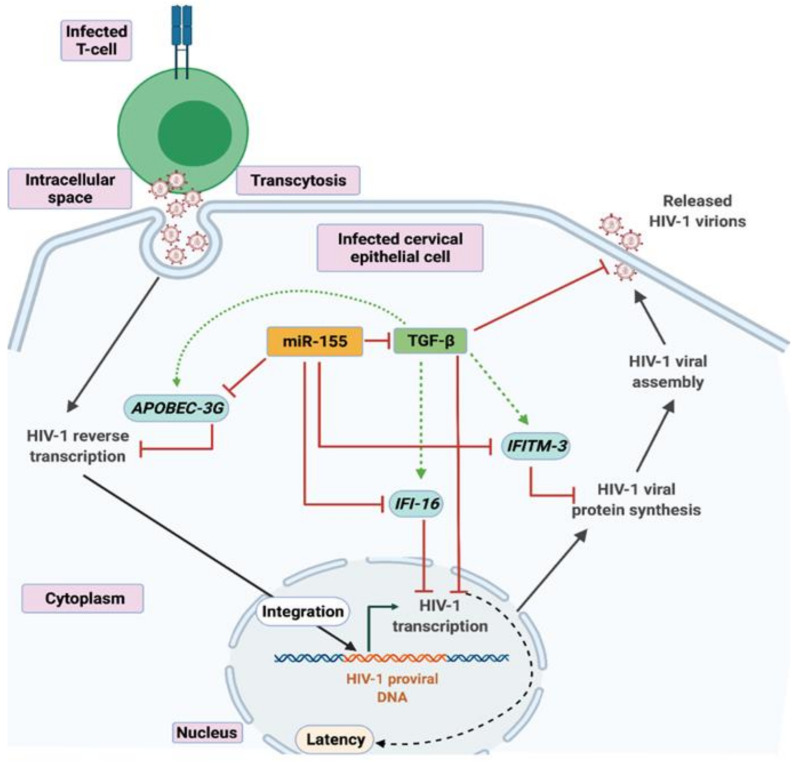
A graphical representation showing the molecular mechanism involved in TGF-β mediated suppression of HIV release in the cervical epithelial cells. HIV-1 infects cervical epithelial cells via cell–cell contact through transcytosis. Reduced TGF-β promotes HIV release in the cervical epithelium which is regulated by miR-155. Inhibition of miR-155 leads to increased TGF-β signaling and host restriction factors—*APOBEC-3G, IFI-16* and *IFITM-3,* resulting in decreased HIV release in the cervical cells. This image is created using BioRender.

In conclusion, to the best of our knowledge, this is the first report which highlights the role of TGF-β as an important regulator in restricting HIV release by the cervical epithelial cells via the suppression of miR-155 (Figure 4). Inhibition of miR-155 increased TGF-β mediated signaling and expression of host restriction factors which could be the likely factors to hamper the virus release by the cervical epithelial cells. These findings indicate a plausible role of TGF-β in promoting HIV latency in the cervical tissues, which needs further investigations. Future studies are warranted where other miRNAs specific to TGF-β signaling could be targeted to provide more evidence on the role of TGF-β protein in regulating HIV release.

## Figures and Tables

**Table 1:** List of miR-155 binding targets on the 3′UTR regions of *APOBEC-3G*, *IFI-16* and *IFITM-3* with seed match of 2–8 bp and their corresponding most optimal miRNA-mRNA duplex hybrid structures. The minimal free energy values (kcal/mol) of the duplex are shown. miR-155 is shown in red and target 3′UTR of the genes are shown in green.

Gene	Position	Minimum Free Energy (mfe) (kcal/mol)	Binding Region	Most Optimal miRNA-mRNA Duplex Hybrid Structures
*APOBEC-3G*	6–30	−19.1	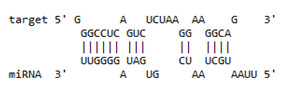	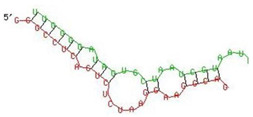
*IFI-16*	122–144	−19.2	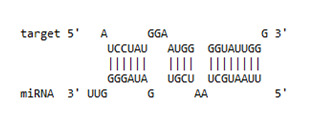	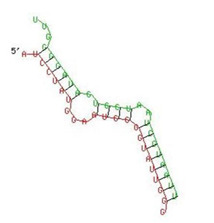
*IFITM-3*	25–47	−17.9	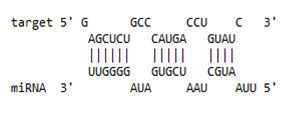	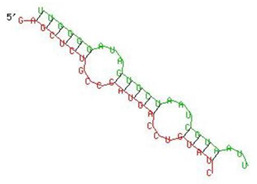

## Data Availability

Data is contained within the results section of this communication.

## Supplementary Materials

The following are available online at https://www.mdpi.com/article/10.3390/v13112266/s1, Figure S1: Validation of absence of T cells from ME-180 co-cultures post Mitomycin-C treatment and washing. Figure S2: Validation of miR-155 depletion in ME-180 cells upon miR-155 inhibitor treatment. Figure S3: Effect of TGF-β neutralization and miR-155 inhibition on HIV GAG gene expression. Figure S4: Effect of miR-155 inhibition alone on the HIV-1 release in the co-cultured ME-180 cells.
