# Supplementary material for: Inhibition of miR-155 Promotes TGF-β Mediated Suppression of HIV Release in the Cervical Epithelial Cells
Source: Viruses. 2021 Nov 12;13(11):2266. doi: 10.3390/v13112266 (PMC8624372; doi:10.3390/v13112266)
Supplement: Supplementary file 1 [file viruses-13-02266-s001.zip › viruses-1454795-supplementary.pdf]

Supplementary Materials:

a.

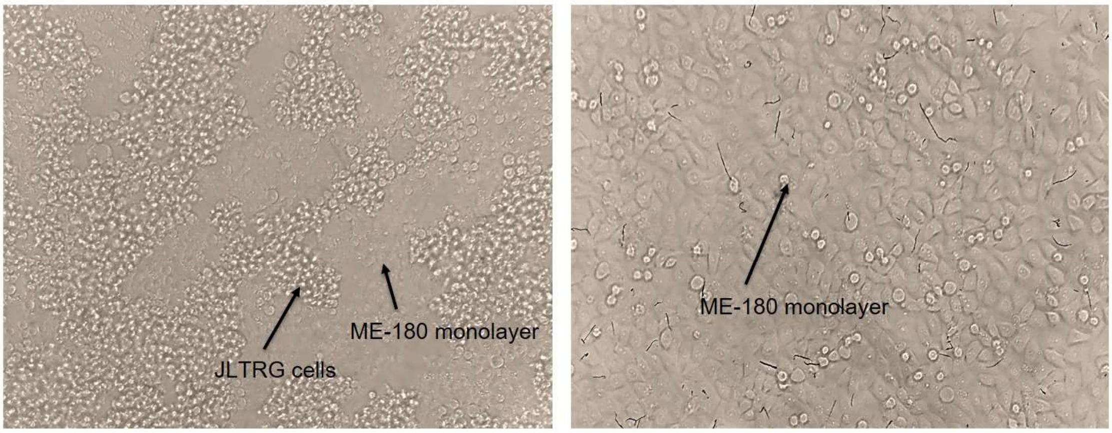

Co-culture of ME-180 cells with HIV-1 NL4.3 infected & mitomycin-C treated JLTRG-R5 cells prior to washing

Co-culture of ME-180 cells with HIV-1 NL4.3 infected & mitomycin-C treated JLTRG-R5 cells devoid of JLTRG-R5 cells after washing

b.

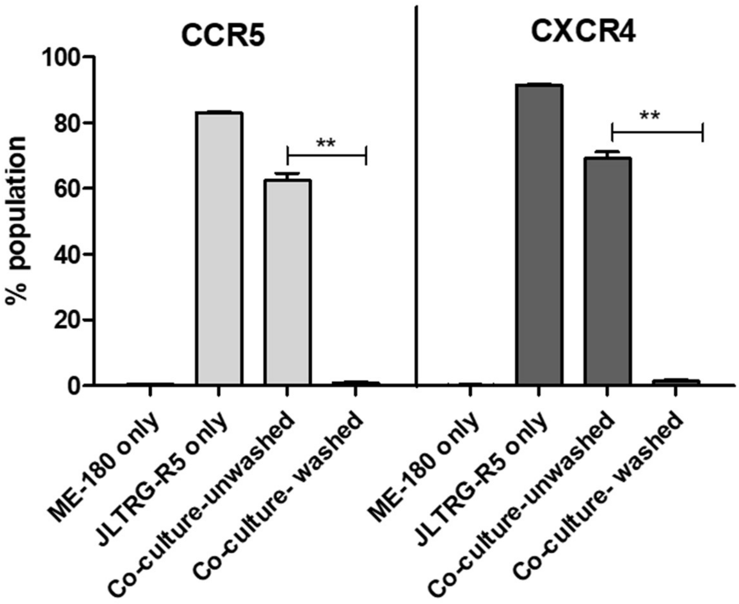

Figure S1. Validation of absence of T cells from ME-180 co-cultures post Mitomycin-C treatment and washing.*(a) Microscopic examination of co-culture of ME-180 and JLTRG-R5 (infected, Mitomycin-C treated) wells before washing image of co-culture after washing thoroughly three times which shows no JTLRG-R5 cells, (b) Expression of co-receptors: CCR5 and CXCR4 in the ME-180 cells co-cultured with infected JLTRG-R5 cells under different experimental conditions using flow cytometry.*

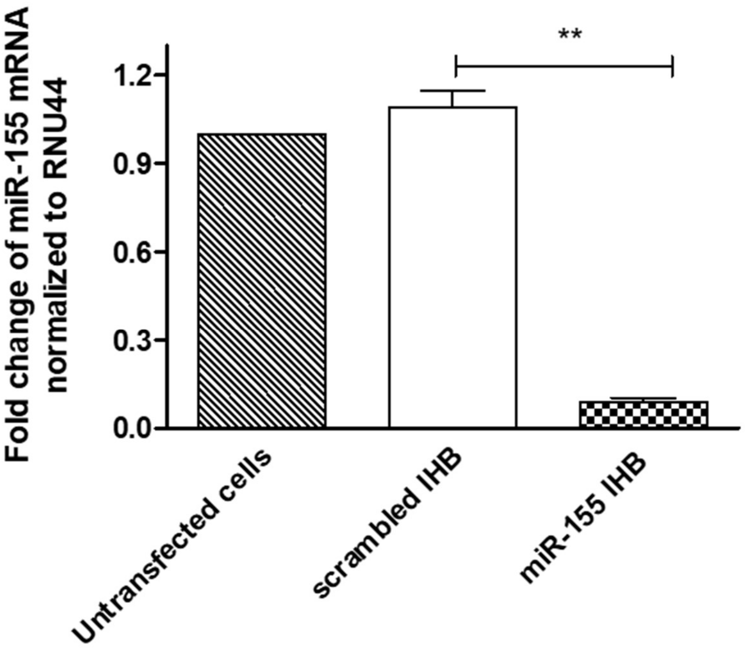

Figure S2: Validation of miR-155 depletion in ME-180 cells upon miR-155 inhibitor treatment. *ME-180 cells were transfected with 100 nM of miR-155 inhibitor, or scrambled miRNA inhibitor (mock) or left untransfected. Expression of miRNA-155 gene was assessed by real-time PCR.*

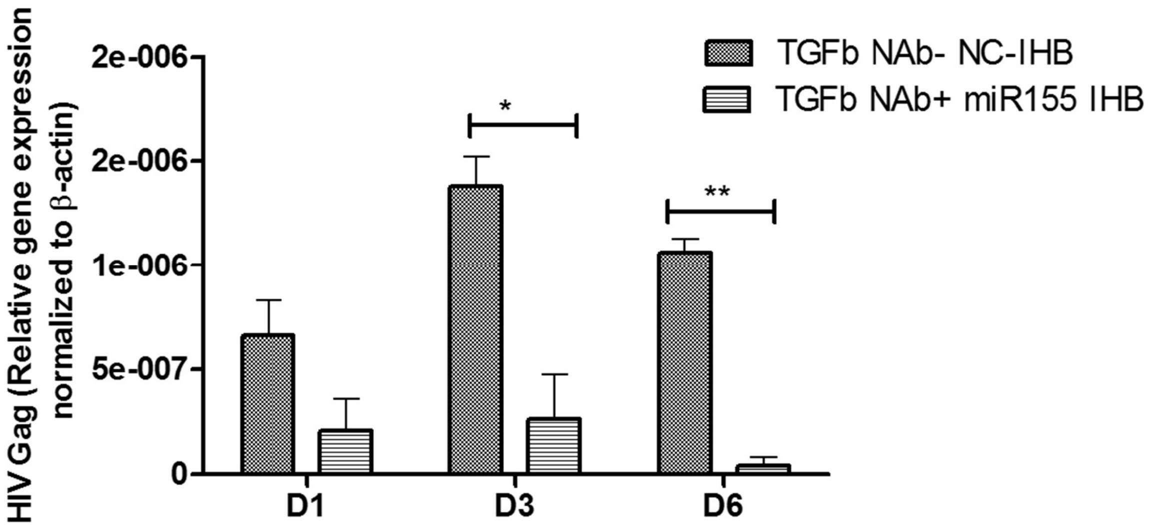

Figure S3: Effect of TGF-β neutralization and miR-155 inhibition on HIV Gag gene expression. *ME-180 cells were transfected with miR-155 inhibitor or scrambled miRNA control and then treated with TGF-β NAb in the miR-155 inhibited cells prior to co-culture. At the indicated time-points GAG gene expression was assessed by Real-time PCR. Gag mRNA expression was normalized to β-actin.*

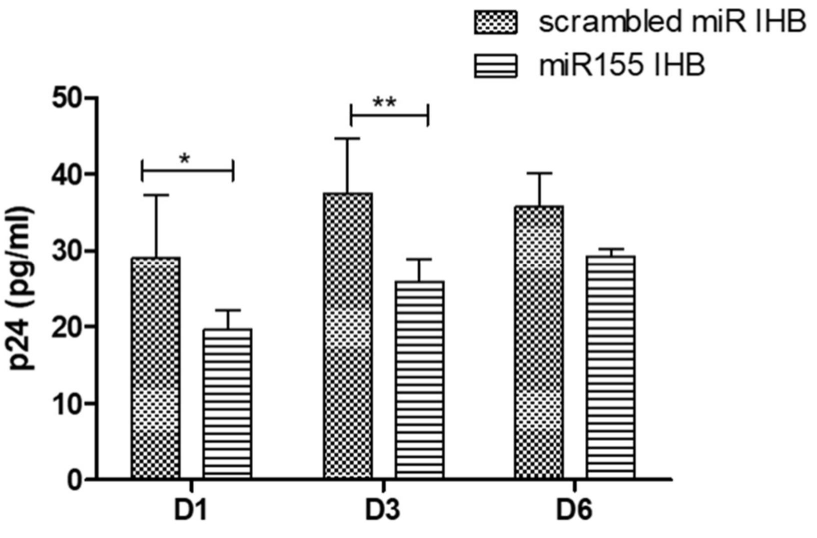

Figure S4: Effect of miR-155 inhibition alone on the HIV-1 release in the co-cultured ME-180 cells. *ME-180 cells were transfected with miR-155 inhibitor or scrambled miRNA inhibitor prior to co-culture with HIV-1 infected JLTRG-R5 cells (mitomycin-C treated). HIV-1 release was assessed by measuring p24 ELISA in the supernatant at the indicated time-points.*
